# Meath Hospital

**Published:** 1831

**Authors:** 


### MEATH HOSPITAL.

ARTHRITIS AND SCIATICA, TREATED BY ACUPUNCTURATION. (Under the superintendence of Mr. HAMILTON, in Dr. GRAVES' wards.)

*Case.* PAT. ROSSETER, ætat. 30, labourer, taken into the hospital November 30th, 1830; complains of pain on motion, and stiffness of both arms and wrists, not very severe, nor very tender on pressure; also severe pain, on motion, a little behind and above the left hip-joint. He walks lamely and with difficulty, not being able to move the thigh, or put his foot firmly to the ground, without great pain. The knees are slightly stiff and painful. None of these parts are red or swollen, and do not give pain while the man remains at rest. They are not worse at night. He attributes them to cold caught from exposure while in a profuse sweat, after a hard day's work, six weeks ago. At first a chilliness came on, and continued for a week, when the shoulders and arms became affected, and for a short time the front of the chest very severely. The pains were erratic, but did not attack the hip or knees till ten days since. Since this attack he sweats often and feels chilly. Bowels regular; appetite and sleep good; pulse full and regular; urine clear, and deposits no sediment.

3d.—In addition to his other symptoms a slight attack of pleurodyny.

℞. Vinum Sem. Colchici, ʒss.

Magnesiæ, gr. x.

Gutta Nig. gt. viij.

Aquæ Cinnam. ℥j. M. sumat ter in die.

Hir. vj. lateri; *Acupuncturation* at the affected part of left hip.

4th.—The needle was pushed in, with considerable pain to the patient, up to the eye in an obliquely-horizontal direction, a few inches above and behind the trochanter, about where the sciatic nerve leaves the pelvis. It was withdrawn after being in twenty-four hours. While in, the part felt sore. Though watched some minutes after its insertion, I could not perceive any action to be produced; the patient himself observed, that he felt it moved. He considers that it has done him good, the pain and tenderness being considerably lessened. His chief cause of complaint now is the left wrist, which is stiff and painful; bowels confined; urine high-coloured, but clear.

Rep. Mist. Colch.

He continued on the use of the colch; sulph. mag. being added merely on one occasion, to open the bowels.

8th.—All his pains much less; and can walk with comparative ease and very little pain to what he had when he came into the hospital; is desirous of having another needle inserted, having experienced so much benefit from the first.

Cont. Colch.

9th.—Pain in the hip returning; the patient is very anxious to have another needle inserted. His other pains less.

Rep. Acupunct. et Mist. Colch.

11th.—The needle was withdrawn, leaving some degree of soreness; his other pains are so trifling that this is almost his only complaint.

Omit. Colch. et sumat Sulph. Quininæ, gr. x.

13th.—Has now no pain any where, and walks extremely well, without the least stiffness or pain.

Although colchicum was taken during the use of the needles, it is evident very little influence can be attributed to this medicine in alleviating the pain in the hip. For though by its means the cure of the other pains was effected, this one, after having been greatly relieved by the first needle, began again to be severe, while the patient was still using colchicum; and a second needle was inserted, at the man's anxious request, with complete relief.

Besides this case, I have seen acupuncturation successful in three others: the first that of Hogan, admitted Sept. 30th, 1830. This man had laboured under inflammation of the anterior crural nerve for two years, and had undergone medical treatment without relief. Four needles were now inserted at intervals; and at the end of a week he was dismissed cured. It is proper to add, that for two days he used Dover's Powder and the warm bath. The second, John Darnford, under Mr. Jones's care, had laboured four months under pain of the hip, with some degree of lameness, and had used blisters and cupping without relief. The second day after admission, two needles having been inserted into the hip, all pain was removed from that part, and he could walk about perfectly well, his only complaint being a pain in the ankle. The last is that of James Toole, in whom one needle removed severe pain in the hip. This patient is under Mr. Bernard's care.

Much talent and ingenuity have been vainly exercised to discover the *modus operandi* of the needle while in the living fibre; any attempt, therefore, on my part, could only end in idle speculation. I trust, however, I shall be excused for venturing to offer a few remarks of a more practical nature; first, on the best manner of inserting the needles; secondly, on the number that should be employed, and the length of time they are to be left in; and, thirdly, on those cases of a rheumatic character in which they are likely to be most beneficial.

1st. It may be observed, that of the above four cases, the two last were much the most striking: the cure occupying only two days in the cases of Darnford and Toole. Many reasons might be brought forward as likely to account for this: the circumstances of the cases, &c. I am inclined, however, to attribute the speedy success of the remedy in a great measure to the different manner in which the needles were inserted. In Darnford's case, Dr. Graves desired the direction of the second needle to be less horizontal, and the next day all pain was removed. In Toole's case, the needle used was so long, and the direction such, as to render it probable that the sciatic nerve was pierced (which Cloquet, I understand, for I could not get his book, considers desirable;) the relief was even more speedy.

In Dr. Renton's hands, acupuncturation has been eminently successful, instantaneous cures having been effected in many cases of long standing and severity, and which had resisted all the other remedies employed. It is difficult to collect from his paper in the Edinburgh Med. and Surg. Journal the precise manner in which he performed the operation. The direction of the needle, however, appears to have been perpendicular, or nearly so, as he lays great stress on the piercing of the muscular fibre, and passes the needle, not up to the eye, but only to the depth of an inch, or an inch and a half, which, were the direction nearly horizontal, would scarcely be deep enough to attain his object. This much is certain, that it was done with a gentle rotatory motion, nor was any pain produced by the insertion of so many as ten needles.

Wishing to satisfy myself on this last head—the absence of pain—I inserted a needle into the centre of the calf of my leg, with a rotatory motion firmly pressing on the top, to about the depth of an inch and a half, the direction being exactly perpendicular. No *pain* was felt; the only feeling being one of great itching. What is curious is, that the needle, after having been in a minute, moved in a circular direction on its own axis; and a numb aching sensation was experienced. It was only left in a couple of minutes, and then withdrawn with some pain and difficulty, as if it had been firmly grasped by the muscular fibres. The leg was the same after as before, and the place of the puncture discovered with difficulty. Now, as pain has not been proved to be necessary to the efficacy of acupuncturation, but will often be a great obstacle to its use in cases where it would be likely to prove a safe, speedy, and efficacious remedy, the insertion of the needle by a rotatory motion—drilling, as it were — being unattended by any pain, must be considered preferable to thrusting it in, a mode which, from Pat. Rosseter's case, we may conceive to be a very painful operation. Dr. Renton's cases, along with the two above-mentioned, would also go far to prove, that the more perpendicular the direction the better, in which case, too, the depth ought to be from an inch to an inch and a half.

With regard to the second point, the number of needles, and the time they are to remain in, there exists great difference of opinion. It is natural to suppose, that if one needle produces any effect, a more powerful one will be produced by many, which is in a great measure confirmed by the great success obtained by Dr. Renton, who used as many as ten in some instances, divided between the hip, thigh, and leg. Dr. Elliotson also uses a considerable number. The former gentleman only allowed them to remain in five or ten minutes; and how he succeeded has been already mentioned. On the other hand, in the Meath, they are left in twenty-four hours; and Dr. Elliotson, in one of his clinical lectures, observes that, 'if needles be merely thrust in, and allowed to remain only a short time, they will in general not be found of much service; they should be left in at least two hours.' It is not easy to reconcile these differences. Most probably more depends on the manner of performing acupuncturation than on any thing else, that the shorter time they are in the benefit should prove to be the greater. If the manner be good, it very likely matters little whether the needles remain in five minutes, or twenty-four hours, as far as the effect is concerned; but it is of great consequence as regards the patient's comfort, who would no doubt sleep better without, than with, nine or ten needles sticking in his body, setting aside the soreness which usually remains after a needle has been in so long. Dr. Elliotson, in spite of having discovered the value of leaving in needles long, appears in some cases to have had more perseverance than success, as he says, 'I once ordered them daily for nine days before I succeeded.' If this, and some other cases given by Dr. Elliotson, are compared with Dr. Renton's, it will be apparent that the remedy must have been differently applied. If performed in Dr. Renton's manner—that is, with half the needle out of the flesh, it is plain it would not be convenient to leave them in long; it is fortunate that there is no necessity, five or ten minutes having proved sufficient.

Lastly, Dr. Elliotson, in considering the cases most likely to be benefited by acupuncturation, divides rheumatism into that attended with a sense of heat, and aggravated by its application; and that in which there is a feeling of coldness, the pain being relieved by warmth. The first of these he judges not likely to be benefited by the use of needles, but in the latter he thinks they will be found to prove very serviceable. But this distinction does not appear to have been acted on by Dr. Renton, as the case of the young woman given by him proves; nor do I recollect it to have been mentioned by Dr. Graves. It is doubtful, therefore, how far it can be considered of importance; and it would probably be better to give the needles a fair trial in all cases of sciatica."*

* Medical Gazette.

